# Desmoplastic Small Round Cell Tumor: Current Management and Recent Findings

**DOI:** 10.1155/2012/714986

**Published:** 2012-03-29

**Authors:** Armelle Dufresne, Philippe Cassier, Laure Couraud, Perrine Marec-Bérard, Pierre Meeus, Laurent Alberti, Jean-Yves Blay

**Affiliations:** ^1^Department of Immunity, Virus and Microenvironnement, Cancer Research Center of Lyon (CRCL), UMR INSERM 1052—CNRS 5286, Leon Berard Cancer Center, 28 Rue Laennec, 69008 Lyon, France; ^2^Medical Oncology Department, Leon Berard Cancer Center, 28 Rue Laennec, 69008 Lyon, France

## Abstract

Desmoplastic small round cell tumor (DSRCT) is a rare and highly aggressive mesenchymal tumor that develops in the abdominal cavity of young men adults. Patients typically present with symptoms of abdominal sarcomatosis. Diagnosis is based on histological analysis of biopsies which typically show small round blue cells in nests separated by an abundant desmoplastic stroma. DSRCT is associated with a unique chromosomal translocation t(11:22) (p 13; q 12) that involves the *EWSR1* and *WT1* genes. The prognosis is particularly poor; median survival ranges from 17 to 25 months, largely due to the presentation of the majority of patients with metastatic disease. Management of DSRCT remains challenging and current schemes lack a significant cure rate despite the use of aggressive treatments such as polychemotherapy, debulking surgery and whole abdominal radiation. Several methods are being evaluated to improve survival: addition of chemotherapy and targeted therapies to standard neoadjuvant protocol, completion of surgical resection with HIPEC, postoperative IMRT, treatment of hepatic metastases with [^90^Y]Yttrium microsphere liver embolization.

## 1. Introduction

Desmoplastic small round cell tumor (DSRCT) is a rare and highly aggressive mesenchymal tumor that was first described as a separate identity in 1989 by Gerald and Rosai [1]; since that time, fewer than 200 cases have been reported in the literature.

DSRCT mainly develops in adolescent and young adults with a strong male predominance; the mean age at diagnosis is approximately 22 years and ranges from 6 to 49 years, the male to female ratio is 4 : 1 [2]. The tumor typically develops in the abdominal cavity, invading the omentum with multiple peritoneal implants involving the diaphragm, splenic hilum, mesentery of small and large bowel, and the pelvic peritoneum. Organ involvement is inconstant and secondary, with liver and lung as two common sites for metastatic disease beyond the peritoneum. Involvement of extraperitoneal organs, such as the testes, ovaries, and pleura has been described in literature. Distant metastasis may occur later [3].

As for of others intraabdominal tumors, patients may be asymptomatic for long periods of time and diagnosis is made when tumor burden is large. Clinically, patients present symptoms of abdominal sarcomatosis such as ascites, abdominal pain and/or distension, constipation or bowel obstruction, vomiting, and weight loss.

Abdominal imaging by ultrasound, computed tomography scan or magnetic resonance imaging reveals multiple peritoneal masses (from millimeter sized nodules to confluent sheets and dozens to hundreds of nodules up to 20 cm or greater). For complete staging, the search for visceral metastasis (hepatic and/or pulmonary) with CT scan is typically used.

## 2. Diagnosis and Staging

Diagnosis is based on histological analysis of biopsies which typically shows small round blue cells in nests separated by an abundant desmoplastic stroma. By light microscopy, tumor cells show immunohistochemical reactivity for epithelial (keratin, epithelial membrane antigen), neural (neuron-specific enolase), and muscle (desmin) markers [4]. DSRCT is associated with a unique chromosomal translocation t(11:22) (p 13; q 12) that involves the *EWSR1* and *WT1* genes [5–7]. The translocation results in a fusion of the 2 genes with expression of an oncogenic chimeric *EWSR1-WT1* protein that acts as a transcriptional regulator that alters gene expression and ultimately permits tumor growth.

DSRCT is a member of the large family of small round cell tumors of childhood, together with PNET (Ewing sarcoma), alveolar and embryonal rhabdomyosarcoma, poorly differentiated synovial sarcoma and rhabdoid tumors.

Disease staging and stage classifications are essential to patient management and allow the comparison of different therapeutic strategies. However, there is currently no validated staging system for DSRCT and using the UICC staging for sarcoma would lead to the classification of nearly all patients as having stage IV disease. Despite aggressive multimodal treatment, median survival ranges from 17 to 25 months, with fewer than 20% of patients achieving 5-year survival.

**Figure 1:**
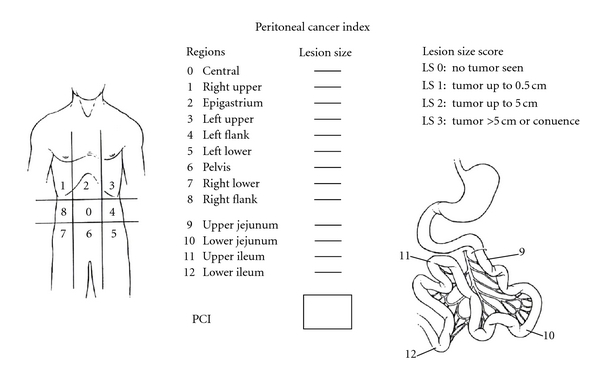


Several staging systems have been proposed to classify peritoneal carcinomatosis. Such a classification is essential to categorise patients and to propose and compare different therapeutic strategies. The staging system currently used is the Peritoneal Cancer Index (PCI), describing 4 stages [8]. Figure 1 presents the PCI.

Recently, investigators at the MD Anderson Cancer Center suggested a new staging system based on the PCI and the presence of liver metastases and extra-abdominal metastases [9].  Table 1 presents this staging system.

Although promising this staging system needs to be validated in larger cohorts and in other institutions.

## 3. Molecular Biology

The unique translocation found in DSRCT involves *EWSR1* and *WT1* genes. *EWSR1* encodes the EWS protein which is a member of the FET family of RNA-binding proteins, while *WT1* encodes a zinc-finger transcription factor. The t(11; 22) found in DSRCT leads to the fusion of the 5 to 9 first exons of *EWSR1* and the 3 last exons of *WT1* [10]. The fusion product is a 59 kDa protein containing the N-terminal portion of EWS, which has strong transactivational properties, and the last three zinc-finger domains of WT1, which acts as an AND-binding domain. The EWSR1-WT1 chimeric protein therefore acts as an oncogenic transcription factor as evidenced by its ability to transform NIH3T3 cells [11]. Several transcriptional targets of the EWSR1-WT1 chimera have been identified such as Platelet Derived Growth Factor A (PDGFA), IL2 receptor *β*, Myeloid Leukemia Factor 1 (MLF1) or Insulin-like Growth Factor 1 receptor (IGF1-R); however, their precise contribution to transformation and their potential as a therapeutic target remain poorly understood.

## 4. Management of Patients with DSRCT

Therapeutic management of DSRCT remains challenging with low efficacy despite the combination of aggressive treatments such as polychemotherapy, debulking surgery and whole abdominal radiation.

Aggressive surgical debulking is the mainstay of the therapeutic strategy. Debulking surgery is defined as definitive removal of at least 90% of the tumor burden. Two retrospective studies of prognostic factors in 32 and 66 patients with DSRCT respectively, identified gross tumor resection as a highly significant predictor of prolonged overall survival [15, 16]. Lal et al. reported a 3-years survival of 58% in patients treated with debulking compared to no survivors beyond 3 years in the nonresection cohort (*P* < 0.00001).

DSRCT is known to be at least somewhat chemosensitive [17] and radiosensitive tumor. The main series evaluating the efficacy of chemotherapy was reported in 1996 by Kushner et al. [18]. Twelve patients were treated with the P6 protocol: 7 courses of chemotherapy with cyclophosphamide (4200 mg/m²), doxorubicin (75 mg/m²) and vincristine (HD-CAV) alternating with ifosfamide (9 to 12 mg/m²) and etoposide (500 to 1000 mg/m²). All tumors responded to HD-CAV, but there were no pathological complete response. Two patients died after chemotherapy (1 Budd-Chiari syndrome and 1 infectious complication). Following response to this induction regimen, tumor resection was attempted; local radiotherapy and myeloablative regimen comprising thiotepa (900 mg/m²) plus carboplatin (1500 mg/m²) with stem cell rescue were administered to 5 and 4 patients, respectively. The median survival time was 19 months for all patients and 22 months for the 7 achieving complete response to chemotherapy. An ongoing trial of NCI evaluates the addition of irinotecan, temozolomide, and bevacizumab to P6 protocol. It is also not clear if such high doses of chemotherapy are any more useful than standard doses of chemotherapy employed in Ewing sarcoma and similar small round cell tumors. Given the poor survival despite these high chemotherapy doses, in the adult population we generally employ lower doses than those described in the Kushner paper.

When such aggressive strategies are not possible, several case reports describing modest activity with anthracyclin-based regimen, trabectedin, or temsirolimus are found in the literature [12–14].

Table 2 presents the efficacy of treatments in relapse setting.

Several authors have advocated the use of hyperthermic intraperitoneal chemotherapy (HIPEC) following optimal debulking in patients with DSRCT. Most patients with DSRCT present with dozens to hundreds of nodules on peritoneum and surgical excision ensuring no microscopic residue is almost impossible to achieve. Efficacy of HIPEC has already been established in peritoneal carcinomatosis secondary to ovarian carcinoma, prolonging survival at the cost of an increased toxicity. In DSRCT, HIPEC has been given as heated cisplatin at a dose of 100 to 150 mg/m². After some case reports, Hayes-Jordan et al. published in 2010 the only one series of DSRCT treated with HIPEC [9, 19]. Retrospective review was performed for 24 patients with DSRCT. Three subgroups were defined according to their treatment and compared: 9 patients received no surgery and were treated with chemo and/or radiotherapy (group 1), 7 patients received debulking surgery (group 2) and 8 patients received cytoreductive surgery and HIPEC (group 3). All patients received neo-adjuvant chemotherapy and some of them abdominal radiation, stem cell transplant and/or immunotherapy. Postoperative chemotherapy with 12 cycles of temozolomide and irinotecan was administered in Aguilera report [19]. The 3-year survival in patients who underwent cytoreductive surgery with HIPEC was 71%, not statistically different when compared with 62% 3 years survival of patients who were treated with surgery alone. The authors explain the lack of statistical significance with the limited size of the sample. Conversely, surgery with or without HIPEC clearly improves survival when compared to patients treated with medical therapy alone (26% 3-years survival). Two more important messages are brought by the article: first, HIPEC seems safer technically in children (rather than in adults); secondly, presence of liver metastasis is not an independent pejorative prognostic factor, but disease outside abdomen is. A prospective phase II study is ongoing, to better define the benefit of HIPEC added to cytoreductive surgery in DSRCT. Overall, data supporting the use of HIPEC in patients with DSRCT is limited and this technique is not recommended for the management of patients with DSRCT outside clinical trials. 

Whole abdominopelvic (WAP) radiotherapy has also been proposed as an adjunct to (complete) surgery with the aim to improve local control. This is based on a report from investigators at the Memorial Sloan Kettering Cancer Center investigating WAP in patients with DSRCT. In this study, patients received induction chemotherapy with the P6 regimen for 7 cycles. Following chemotherapy and maximal surgical debulking, 21 patients received external beam radiotherapy to the whole abdomen and pelvis to a dose of 30 Gy plus a radiation boost to remaining tumor sites for patients with gross residual disease. WAP was associated with significant gastrointestinal and haematological toxicity (requiring red blood cell transfusion and GCSF support in some patients). Long-term toxicity consisted of small bowel obstruction (7/21 patients) and ureteral stenosis (2/21 patients). Furthermore, most of the patients relapsed (16 of 21, 76%) and eventually died of their disease while one patient died of acute leukemia while in complete response. All of the 16 relapses were seen in the radiation field. Overall only 2 patients (10%) were alive and disease-free at last follow-up, data that appear no different than other case series in the literature. 

More recently, Pinnix et al. reported a series of 8 patients treated with whole abdominopelvic Intensity-Modulated Radiation Therapy (IMRT) after neoadjuvant chemotherapy and debulking surgery (and HIPEC for 7 patients) [20]. They conclude that postoperative IMRT is feasible and well tolerated after aggressive surgery with no grade 4 digestive symptoms, red-cell transfusions in only 2 patients and grade 4 cytopenia in only 1 patient. No other cytopenia was noted. Among these 8 patients, only 1 did not relapse after 20 months follow-up. Based on these reports, WAP radiotherapy (WAP-RT) appears feasible in patients with DSRCT, but is associated with significant toxicity and limited efficacy. Again, with no survival signal in the studies to date, it is difficult to recommend this modality outside of a clinical trial. 

Recently, Subbiah et al. reported the case of a young patient with hepatic metastasis of DSRCT resistant to chemotherapy successfully treated with [^90^Y]Yttrium microspheres given by hepatic artery embolization with evident metabolic response on PET-CT [21]. Given the finding of peritoneal disease in most patients, it seems therapy by hepatic artery infusion will have a very limited place in treatment of DSRCT. 

## 5. Conclusion 

Despite its rarity, several new procedures have been tested in DSRCT in this particularly severe tumor affecting children, without evidence of clinical utility. The combination of Ewing-sarcoma-based polychemotherapy and debulking surgery represent the standard of care as of early 2012. Not surprisingly, those patients having successful surgical debulking and responsive disease to chemotherapy appear to have the best outcome compared to groups of patients who do not achieve both favorable outcomes. The impact of new techniques such as HIPEC or IMRT needs to be clearly defined, ideally in the context of prospective randomized clinical trials since the retrospective data to date give no sense of a positive survival signal. Whole genome sequencing of DSRCT is ongoing to identify mutations, single nucleotide polymorphisms or copy number changes associated with these tumors to explore pathogenesis and open medical therapeutic possibilities. 

## Figures and Tables

**Table 1:** 

Stage	PCI	Liver metastasis	Extraabdominal metastasis
I	<12	No	No
II	>12	No	No
III	Any PCI	Yes	No
IV	Any PCI	Yes or no	Yes

**Table 2:** 

Author	Drug	Number of cases	Benefit
Thijs et al., 2010 [12]	Temsirolimus	1	PFS 40 weeks
Lopez-Gonzales, 2011 [13]	Cisplatin-Campto trabectedin	1	PDPFS 8 months
Chao, 2010	Imatinib mesylate	2	PFS 0.2 and 1.1 months
Mrabti et al., 2011 [14]	Anthracyclin	1	—
Outc's observatory ASCO 2010 #10097	Sunitinib	2	PFS 2 and 6 months
Outc's observatory ASCO 2010 #10097	Sorafenib	2	PFS 3 months: stop at 3 months for toxicity
